# Sex‐Specific Effects of Social Environment on Behaviour and Their Correlations in *Drosophila melanogaster*

**DOI:** 10.1002/ece3.71261

**Published:** 2025-04-25

**Authors:** Erin L. Macartney, Samantha Burke, Patrice Pottier, Zina Hamoudi, Chloe Hart, Radiah Ahmed, Yong Qi Lin, G. Gregory Neely, Szymon M. Drobniak, Shinichi Nakagawa

**Affiliations:** ^1^ Evolution and Ecology Research Centre, School of Biological, Earth and Environmental Sciences University of New South Wales Sydney New South Wales Australia; ^2^ Charles Perkins Centre, School of Life and Environmental Sciences The University of Sydney Sydney New South Wales Australia; ^3^ Institute of Environmental Sciences Jagiellonian University Krakow Poland; ^4^ Department of Biological Sciences, Faculty of Science University of Alberta Edmonton Canada

**Keywords:** behavioural plasticity, behavioural syndrome, environmental enrichment, insect, mating, sex differences, social deprivation

## Abstract

Environmental and individual experiences can result in immediate and persistent changes in behaviour. Often, such effects are also sex‐dependent. Intraspecific interactions can be one of the most important environments an individual faces. Such social interactions are expected to affect a suite of behavioural traits and their correlations. Here, we used *Drosophila melanogaster* and high‐throughput automated behavioural phenotyping to determine how social environment (group mixed sex, group single sex, and social isolation) and sex interact to affect basic behaviours (exploration, movement within a y‐maze, and habituation to a startle) that likely underlie more complex behaviours such as mate searching and foraging. We show that such behaviours and some behavioural correlations are indeed context‐ and sex‐dependent. Males tended to show greater exploration, while females were more likely to show a habituation response to startle. Males and females from the mixed sex and isolated treatments showed opposite exploratory behaviour in the Y‐maze, and social treatment interacted with sex to affect the rate of habituation to a startle. Females also tended to have slightly stronger trait correlations compared to males. These results show that social environment and sex can play a significant role in shaping behaviour in *Drosophila melanogaster* . Our study provides insights into how the type of social stimulation and sex can interact to affect behaviours that are important in forming critical behaviours related to foraging and mate searching.

## Graphical abstract

Behavioural traits are plastic in response to the social environment. The perceived amount of sex‐linked behavioural contexts modifies the expression of activity‐related traits in a sex‐specific manner.

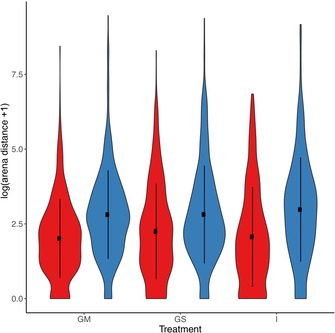

## 1 Introduction

Individuals are constantly sensing their surrounding environment and adjusting their behaviour accordingly (Dingemanse and Wolf 2013; Snell‐Rood 2013). In many taxa, multiple sensory modalities, including vision, audition, olfaction, tacticion, gustation, and nociception, have evolved to detect the environment, often through ‘cross‐modal’ interactions (Shimojo and Shams 2001; Sur et al. 1990). Using these senses, environmental information is conveyed to neuronal networks that trigger behavioural responses (Snell‐Rood 2013). Behavioural responses can be ‘activational’, where an individual immediately reacts to a stimulus (e.g., hiding from a predator) (Snell‐Rood 2013), or persistent, resulting in long‐term behavioural changes (Maleszka 2016; Sinn et al. 2008). As behaviours can be consistent within individuals, forming the basis of personalities and behavioural syndromes (Dingemanse and Dochtermann 2013), persistent behavioural changes due to the environment can be seen as integral parts of the repeatable behaviour machinery (Kempermann 2019).

A paradigm that is often used to test the stimulation of multiple sensory pathways on behavioural plasticity is ‘environmental enrichment’; an assumption that specific changes in the complexity of the surrounding environment can enhance animals' natural behaviours (Freund et al. 2013; Hebb 1949; Kempermann 2019; Macartney et al. 2022). For example, complex and novel ‘enriched’ environments require the use of multiple sensory pathways that can result in persistent changes in brain structure and function (Freund et al. 2013; Kempermann 2019; Kozorovitskiy et al. 2005; Mohammed et al. 2002; Singhal et al. 2014). Increases in sensory stimulation can alter brain characteristics such as brain size and weight, while sensory deprivation can disrupt normal neuronal functioning (reviewed in Baroncelli et al. 2010; van Praag et al. 2000). These changes in brain structure and function can manifest as changes in many behaviours, including exploratory behaviours, learning, and memory (Gardner et al. 1975; Heisenberg et al. 1995; Margulies et al. 2005; Nithianantharajah and Hannan 2006). Resulting modifications of behaviour and physiology may have measurable impacts on animal survival and reproduction, contributing to the fitness of captive or farmed species (Carlstead and Shepherdson 1994; Arechavala‐Lopez et al. 2022; Zhang et al. 2022).

Comparing animals reared in typical laboratory vs. enriched conditions may be argued to be of little importance—after all, it is the enriched environment that should reflect the natural environment of a species, and thus such comparisons may have questionable applications in terms of their biological adequacy. However, any differences observed in such contexts provide valuable insights into evolutionary and ecological processes that drive the evolution of animal behaviours linked to environmental enrichment (Newberry 1995). It may also inform how wild animals respond to changes in the complexity of their natural environment, an important pattern because of the known feedback between environmental enrichment and the plasticity of the nervous system (Nithianantharajah and Hannan 2006). Consequently, such comparisons could expose feedbacks maintaining optimal levels of intraspecific interactions in natural contexts.

Arguably, intraspecific interactions are one of the most important environments an individual interacts with. Social interactions provide individuals with a range of stimuli and challenges, such as opportunities for mating, communication, or competition. Indeed, studies on taxonomically diverse species have shown that social interactions can change brain functioning (Arechavala‐Lopez et al. 2022; Cummings et al. 2008; Ellis and Carney 2010; Gardner et al. 1975; Yeh et al. 1996). Furthermore, inter‐sexual interactions to assess mate quality prior to and during mating often require the use of many senses and neural pathways, which can then affect behaviour (Hollis and Kawecki 2014; Maggu et al. 2022; Kurtovic et al. 2007; Lin et al. 2016; Mak et al. 2007; Agrawal et al. 2014; Houde 1987). For example, studies on *Drosophila melanogaster* have shown that expression of behaviour‐related genes in female brains can change in response to courtship cues from males (Immonen and Ritchie 2012) and that males and females can show sex‐specific gene expression in response to mating, which may then correspond to behavioural changes (see Mank et al. 2013). Intra‐sexual interactions, such as assessing competitors, are also likely to affect behavioural plasticity but may have different effects compared to inter‐sexual interactions (Dankert et al. 2009). Additionally, individuals that lack any form of social interaction can often show atypical behaviour, potentially due to stimulus deprivation and disrupted development (Dankert et al. 2009; Sethi et al. 2019).

One of the important social contexts in animal behaviour is related to mating—as such inter‐sexual interactions can have not only overall, but also sex‐specific effects on individuals. For example, the effects of mating have been shown to differentially affect gene expression in the head and thorax in female *D. melanogaster* compared to males (Fowler et al. 2019), and such differences in gene expression may then correspond to differences in behaviour (Bath et al. 2017; Carvalho et al. 2006; Isaac et al. 2010). Such sex‐specificity also extends to cross‐trait correlations: Han et al. (2015) showed that the correlation between boldness behaviours under different mating environments differed between males and females in the water strider ( *Gerris gracilicornis* ). Similarly, Videlier et al. (2019) found that the correlation between resting metabolic rate and locomotor behaviour was sex‐specific and environment‐dependent in *D. melanogaster*. Thus, social environments may alter behaviours and behavioural correlations differently for males and females.

Despite considerable research effort, the effects of the social environment remain a poorly understood aspect of the complex interplay between environment and behaviour. In particular, we only have fragmentary knowledge on the impacts of social environment on overall behavioural responses, especially in the context of sex‐specific effects. To examine how intraspecific social environment affects male and female behaviour, we determined the effects of varying levels of social environment enrichment and sex (both direct and interactive) on three basic behavioural traits related to exploration, stress habituation, and memory. We also determined if these traits were correlated and if the strength of the correlations was affected by social environment and sex. To do this, we housed adult *Drosophila melanogaster* of both sexes in isolation (I) or in either group mixed sex (GM) or group single sex (GS) vials of equal density. We assayed them using a high‐throughput phenotyping setup to measure several proxies of exploratory and memory behaviours.

Similar behaviours have already been shown to have modified responses under environmental enrichment (van Praag et al. 2000), so we predicted that (i) isolated individuals (i.e., deprived of any form of social stimulation) would show reduced behavioural responses compared to individuals exposed to social stimulation, (ii) inter‐sexual interactions (i.e., being held in mixed sex groups) would result in the highest sensory stimulation which may manifest in the strongest behavioural responses, and (iii) males and females would respond differently to the social environment (i.e., interaction between social environment and sex). The latter could result from the fact that mating can also result in various physiological changes, such as reduced female receptivity to mating after exposure to male seminal fluid proteins. Because female movement is attractive to males, this may correspond to altered behaviours such as reduced female exploration relative to males (Laturney and Billeter 2014; Tompkins et al. 1982), either due to the detrimental effects of seminal fluids, or a tendency to avoid further insemination. Importantly, assuming a socially enriched environment as a biological default, our predictions should conceptually be seen as reductions in observed responses when exposed to the suboptimal social environment.

Phenotypic traits only exceptionally can be considered as isolated, univariate characters. To this end, we also predicted that the measured behavioural traits would be positively correlated but that the strength of the correlations would be sex‐ and social environment‐specific, potentially reflecting differing roles these behaviours play in inter‐individual interactions between the sexes. Specifically, we predicted looser correlations in socially deprived environments, in line with the expectation of such deprivation exerting novel, stressful pressure (Wood and Brodie 2015). Expanding the focus to correlations reflects the established view that phenotypic traits are not isolated and that patterns of trait covariation may have considerable impacts on their evolutionary potential, including in social and mating contexts (Prokop and Drobniak 2016; Oh and Shaw 2021). Arguably, integration of various movement/exploration related traits would be stronger in males (as their reproductive success should depend more on such behaviours) and in contexts providing increased social stimulation (which, in turn, could be seen as a form of behavioural plasticity). Understanding such context‐ and sex‐specific effects on behavioural plasticity and correlations is expected to provide insights into how social stimulation and complexity of the social environment can change suites of behaviours that underline critical functions, such as foraging or mate searching. Identified patterns would also inform future efforts to decipher evolutionary constraints present in behavioural syndromes stemming from trait integration (Garamszegi and Herczeg 2012).

## 2 Methods

### 2.1 Study Animals

We used Canton‐S wild‐type *Drosophila melanogaster* reared with overlapping generations at the Charles Perkins Centre, the University of Sydney. We did not require ethics approval for use of these study animals. Stock flies were kept at 25°C, 65% humidity, and a 12:12 light: dark cycle. Experimental flies (see below) were reared in the same conditions but at 60% humidity. All fly stocks and experimental flies were reared on a standardised food medium consisting of 1625 mL molasses, 325 g yeast, 1000 g cornflour, 150 mL propionic acid, 300 mL Nipagen, 150 g agar, and 24,200 mL water.

The larvae of the experimental flies were reared in a standardised density (~50 per vial) in 55–65 mL of food medium. Adult flies were collected as virgins (< 8 h post adult eclosion) and randomly allocated to adult treatment vials. We had three treatments: isolated individuals (I), group single sex (GS), and group mixed sex (GM). Isolated (I) treatments had one single male or one single female per vial, the GS treatment had either 10 males or 10 females per vial, and the GM treatment had five males and five females per vial. Therefore, while males and females were housed together in the same vial for the GM treatment, we had six treatments and sex combinations to be used in behavioural assays.

The flies eclosed in six batches spread across 3 weeks (two batches per week). For each batch, we had 40 I male and I female vials, 8 GS male and GS female vials, and 12 GM vials (see below for sample size). Flies from each batch were transferred into their treatment vials at the Charles Perkins Centre and were then delivered to the School of Biological, Earth, and Environmental Science, University of New South Wales, Sydney, for housing and completion of the behavioural assays. Each batch was split into two ‘sessions’, where half the batch was housed in an incubator where the 12 h light cycle started at 9 am and the other half of the batch was stored in an incubator where the 12 h light cycle started at 1 pm. This was so we could assay all flies from one batch on a single day (i.e., in a morning session and an afternoon session) while standardising the circadian rhythm so that all sessions were conducted in the ‘morning’ activity period for the flies and avoid their mid‐day low activity period. Flies were housed in their treatment vials for 8 days (flipped into new vials with fresh food on Day 3 and 6) prior to the behavioural assays.

### 2.2 Behavioural Assays

The assay methods are based on the detailed methods reported in Macartney et al. (2022). Briefly, we used high‐throughput automated tracking units produced by Zantiks (Cambridge, UK). These units are designed to track small‐sized animals where each unit consists of (i) an experimental chamber where the animals are placed (see below for details of the ‘plates’ that the individuals are loaded into prior to being placed in the unit chamber), (ii) a camera that tracks the animals, and (iii) a computer controlling the units. All units were programmed to maintain 25°C in the chamber.

Using these units, we conducted three different behavioural assays: (1) locomotion tracking where the overall movement of individuals was measured within a 1 cm deep and round arena, (2) startle response to a light‐off startle (also conducted in the same 1 cm deep and round arena as the locomotion assay), and (3) exploration in a Y‐maze (see Macartney et al. 2022 for more details about the arenas). The locomotion tracking assay ran for 30 min (including a 10‐min habituation period), and then the distance travelled by each individual was recorded across three intervals that were 10 min each. The light‐off startle response recorded the distance travelled in a 1‐s interval following three consecutive 15‐ms light‐off pulses, allowing us to measure habituation across the pulses. The y‐maze assay recorded ‘trigrams’ of the direction flies travelled between arms (e.g., RRR, LLL RLR, LRL, etc., where *R* = right, L = left) (see Macartney et al. 2022.

In all assays, the arenas used had limited area and were restricted in size compared to spaces routinely experienced by fruit flies in their environment. However, existing evidence suggests that such a spatially restricted arena is still a valid measurement tool that can quantify repeatable and clearly defined components of an animal's behaviour. Flies placed in such restricted spaces exhibit increased levels of initial locomotory activity but return to velocities and distances covered within several minutes of acclimation (Xiao and Robertson 2015). Moreover, independent of arena size, fruit flies seem to have a universal tendency to border exploration (Soibam et al. 2012). Studies using arenas other than circular similarly evidenced good agreement between less and more restricted behaviour assaying paradigms: in linear, narrow arenas, fruit flies exhibited similar levels of focal behaviour compared to unrestricted, two‐dimensional settings (Stern et al. 2019; Seidenbecher et al. 2020; dos Santos et al. 2024).

For each session (i.e., morning and afternoon session per batch), we assayed 84 flies (14 flies per treatment and sex combination, i.e., 28 per experimental group). In total, we assayed 186 flies per batch and 1008 flies in total (168 flies per treatment and sex combination; see below for final sample size after accounting for deaths and lack of movement detected within arenas).

At the start of each session, all flies were anesthetised by briefly submerging vials in a bucket of ice. All flies from each treatment and sex combination were then tipped into separate Petri dishes. Fourteen flies from each treatment and sex combination were randomly selected from their respective Petri dishes and aspirated into either a 48 well‐plate (only 42 wells were filled) or into a y‐maze plate; y‐mazes consisted of three plates with 15 mazes per plate and we filled 14 mazes per plate (see Macartney et al. 2022 for further details). The order that flies from each treatment and sex combination were transferred into the well‐plate or y‐mazes was predetermined using a random‐number generator so that flies from each treatment and sex were randomly distributed across the plates. Flies were then given 10 min at 25°C to recover and were transferred into four different assay units: one for the well‐plate with all 42 flies and three units for each of the three y‐maze plates. The unit with the well plate always recorded locomotion first, followed by the light‐off startle response.

After each unit completed the assay, the well‐plate and y‐maze plates were briefly placed in a −25°C freezer until the flies were anesthetised again. All individuals from the well‐plate were then transferred to three clean y‐maze plates, and the individuals from the y‐maze plates were transferred to a clean well‐plate. Each location in the well‐plates and y‐mazes always corresponded to each other (i.e., individuals in the first well were always transferred into the first y‐maze) so that we could keep track of individual flies when transferring them between plates (see Macartney et al. 2022). Flies were then given another 10‐min recovery period at 25°C, then were transferred back into the units to run the assays (i.e., individuals that were previously in the well‐plate and had experienced the locomotion and light‐off startle response assays were now in the units for the y‐maze assay and vice versa). Flies were then discarded after the assays.

The division of assays into batches and the organisation thereof was dictated by the timing of each assay and was organised to allow for the longer y‐maze trail to run concurrently with the shorter general locomotion assays. Assays were also grouped into morning and afternoon sessions deliberately to avoid the mid‐day “siesta” period of lower activity of fruit flies. Since flies were always randomly assigned to the morning/afternoon sessions and all other aspects of behavioural assays were uniform across sessions, we do not expect any bias resulting from two differently timed assay batches.

### 2.3 Data Analysis

Each unit produced a datasheet per assay run. All datasheets were labelled with a unique ID, meaning that we could match each datasheet to individuals, their social treatment, and sex. All data was cleaned and analysed in the R environment (version 4.2.2) using RStudio. All data and code can be found on GitHub (https://github.com/elmacartney/Droso_social_env_behaviour) and in a permanent Zenodo repository (https://zenodo.org/doi/10.5281/zenodo.13347772).

First, we conducted univariate models using the *lme4* package (Bates et al. 2015). Each model included treatment and sex as fixed effects and the treatment × sex interaction. Batch ID and individual ID were included as random effects in all models. Individual ID was included as a random effect in the locomotion and startle‐response assay (habituation) as observations were conducted over three time intervals, and individual ID was included as a random effect in the y‐maze analysis as an observation‐level random effect to account for over‐dispersion. Both locomotion and light‐off startle response data were analysed as linear mixed‐effects models with a Gaussian distribution where the total distance travelled (log‐transformed to improve residual normality) was the response variable. The startle‐response assay also included a three‐way interaction between startle number (i.e., startle 1, 2, or 3), sex, and treatment, as well as two 2‐way interactions between startle number and sex, and startle number and treatment. The y‐maze data was originally analysed using a three‐way interaction between the type of trigram response, sex, and treatment (see Supporting Information), but was also analysed using two separate generalised linear mixed effect models with a Poisson distribution where the response variable was either the number of alternating (e.g., LRL or RLR) or sequential trigrams (LLL, RRR). We did not include partial trigrams in the analysis due to more types of partial trigrams (LLR, RRL, RLL, LRR), thus biasing the number of partial trigrams relative to alternating and sequential trigrams. The Poisson error structure and log‐link were chosen as the most appropriate for count data resulting from y‐maze trials.

For all analyses, we removed flies that were not detected to show any movement (see below for final sample sizes). These flies were removed as we cannot differentiate between the ‘true effects’ of the fly not moving or due to issues with the machine detecting movement. However, we did find that a large majority of flies did not show a startle response to the light‐off stimuli, suggesting that only some flies were sensitive to the startle. We, therefore, conducted an additional analysis using a generalised linear mixed‐effects model with a binomial distribution (1 = the fly showed any startle during the assay, 0 = no startle detected) to assess if there were any treatment or sex effects on which flies showed a startle at all or not. This model included the main and interactive effects of sex and treatment as well as batch as a random effect.

Test statistics and *p*‐values for all models were calculated using the *Anova* function from the *car* package (Fox and Weisberg 2019). We calculated the percentage of variation explained by differences between individuals and differences between batches out of the total variation in the model for each behavioral response using the *rptR* package (Stoffel et al. 2017), which implements ICC (intra‐class correlation) and marginal *R* ^2^ via mixed‐effects models (Nakagawa et al. 2017; Nakagawa and Schielzeth 2013).

To explore multivariate (multi‐response) patterns of correlations between pairs of variables, we have fitted multi‐response mixed models to our data using the *MCMCglmm* R package (Hadfield 2010). This package's use was dictated by its ability to accommodate multi‐response models exhibiting different underlying error structures and by its ability to model both heterogenous random and residual variance effects. The model had a similar fixed effects structure as described above. However, it fitted a heterogenous (split by treatment groups and by sex) covariance matrix for the included behavioural traits, which resulted in a block‐diagonal covariance structure across all modelled traits and treatments. The model was run for 100,000 iterations, with the ‘burn‐in’ period of 20,000 iterations and posterior sampling every 80 iterations. We used uninformative inverse‐Wishart priors for all (co)variance parameters. In its final version, the model included three response variables (log‐transformed locomotory activity, assumed to be normally distributed; the number of repetitive turns in the y‐maze assay, assumed to be Poisson‐distributed with a log‐link function; startle response magnitude, assumed to be normally distributed). Estimated (co)variances were used to derive treatment‐ and sex‐specific correlations between pairs of traits.

Due to some deaths and losses during the assay period or no movement detected during the assays, our final sample sizes were reduced and varied between assays. *N* = 930 flies for the locomotion assay, *N* = 694 for the y‐maze assay, *N* = 930 for the startle response assay when analysed with a binomial distribution (assessing if flies showed a startle response at least once during the assay), *N* = 188 for the startle response assay where flies showed a startle response across all three startles (used to test for habituation). See figure legends for treatment and sex sample sizes for each assay.

## 3 Results

### 3.1 Effects of Social Environment and Sex on Average Behavioural Traits

**FIGURE 1:**
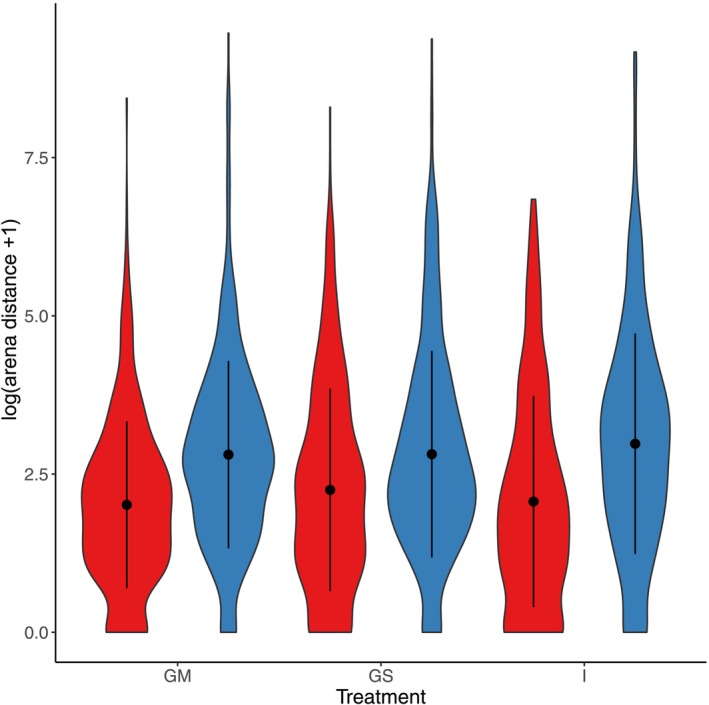
Violin plot showing treatment and sex effects on the logged arena distance (+1) that the flies travelled during the locomotion assay. Red = females, blue = males, GM = group mixed sex, GS = group single sex, I = isolated individuals. Point and line in black represent mean ± SD Samples sizes were *n* = 157 for GM females, *n* = 154 for GM males, *n* = 157 for GS females, *n* = 160 for GS males, *n* = 148 for I females, and *n* = 154 for I males.

We show that males travelled larger distances during the locomotion assay than females (Figure 1; Table 1). We did not detect any effect of social treatment nor a treatment × sex interaction in the locomotion assay (Table 1). Differences between individuals explained 41.89% of the total variation in locomotion, and differences between batches explained 5.66% of the total variation in locomotion.

**TABLE 1:** The effects and interactions of sex and treatment on locomotion, startle response (binomial where individuals showed at least one startle, and habituation where individuals showed a startle response across all three light‐off pulses), Y‐maze alternations and repetitions. Startle response includes an interaction with startle number.

	Locomotion			Startle (binomial)			Startle (habituation)			Y‐maze alternations			Y‐maze repetitions		
	*χ* ^*2*^	df	*p*	*χ* ^*2*^	df	*p*	*χ* ^*2*^	df	*p*	*χ* ^*2*^	df	*p*	*χ* ^*2*^	df	*p*
Treatment	1.78	2	0.41	**19.98**	2	**< 0.001**	4.41	2	0.11	2.97	2	0.22	4.57	2	0.10
Sex	**90.07**	**1**	**< 0.001**	2.18	1	0.14	3.22	1	0.07	**83.74**	**1**	**< 0.001**	**214.85**	**1**	**< 0.001**
Startle number	—	—	—	—	—	—	**20.68**	**2**	**< 0.001**	—	—	—	—	—	—
Treatment × sex	3.49	2	0.17	2.15	2	0.34	5.50	2	0.06	**93.52**	**2**	**< 0.001**	**156.34**	**2**	**< 0.001**
Treatment × startle number	—	—	—	—	—	—	0.40	2	0.82	—	—	—	—	—	—
Sex × startle number	—	—	—	—	—	—	**6.93**	**1**	**0.01**	—	—	—	—	—	—
Treatment × sex × startle number	—	—	—	—	—	—	**6.54**	**2**	**0.04**	—	—	—	—	—	—

*Note:* The *p*‐values in bold were deemed statistically significant at the Type I Error threshold of 0.05.

**FIGURE 2:**
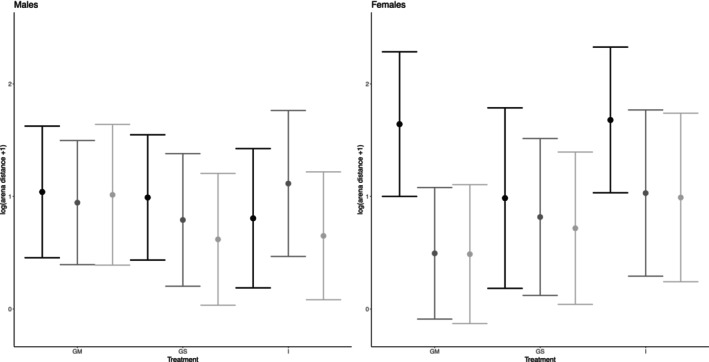
Mean ± SD plot showing treatment and sex effects on the logged arena distance (+1) that the flies travelled after each light‐off startle within treatments and sex. GM = group mixed sex, GS = group single sex, I = isolated individuals. Dark to light represents first through to third startle. Sample sizes for the number of individuals that showed at least one startle (binomial analysis) were *n* = 157 for GM females, *n* = 154 for GM males, *n* = 157 for GS females, *n* = 160 for GS males, *n* = 148 for I females, and *n* = 154 for I males. Sample sizes for the number of individuals that showed a startle across all three startles (habituation analysis) were *n* = 16 for GM females, *n* = 27 for GM males, *n* = 26 for GS females, *n* = 33 for GS males, *n* = 42 for I females, and *n* = 44 for I males.

For the startle response, we detected a main effect of social treatment on whether the flies responded at least once to the stimuli (i.e., binomial analysis) (Table 1). In this analysis, we found that isolated individuals (I) were more likely to show a startle response relative to the group mixed sex (GM) individuals (Z = 3.90, *p* < 0.001) and group single sex (GS) individuals (Z = 0.69, *p* = 0.01). We did not find significant differences in the startle response between GM and GS individuals (Z = 1.65, *p* = 0.10). When only analysing the individuals that showed a startle across all three startles to test for a habituation response, we detected a significant three‐way interaction between treatment, sex, and startle number (Figure 2; Table 1). We show that females exhibit greater habituation to the startle across the three startles. Females from the group mixed sex (GM) and isolation (I) treatments also showed a much larger reaction to the first startle, then displayed habituation (i.e., reduced reaction) to the following startles (Figure 2). Group single sex (GS) females also appeared to show habituation but to a lesser degree (Figure 2). Males did not appear to show strong habituation to the startles overall, except for a trend towards reduced reactions with each consecutive startle in the GS treatment, similar to the habituation response shown in GS females. Differences between individuals explained 14.69% of the variation in the startle response, and there was no detectable variation due to batch (< 0.001%).

**FIGURE 3:**
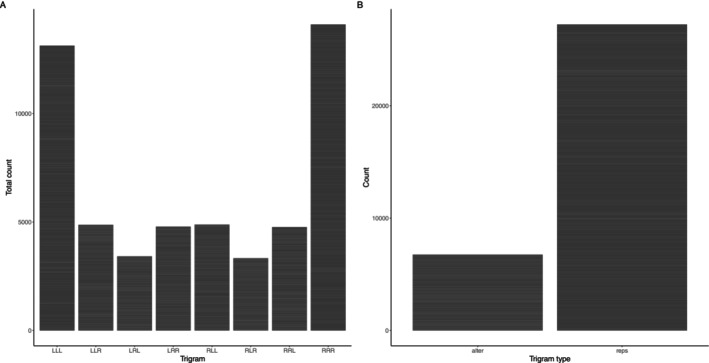
Bar charts showing (A) the number of trigrams the flies performed during the y‐maze assay and (B) the broad trigram types (i.e., alternation or repetition trigrams). Alternations = LRL, RLR, repetitions = LLL, RRR. Note that partial trigrams (LLR, LRR, RRL, RLL) were excluded in panel B due to the bias in the number of trigram categories, which would inflate the number of counts.

**FIGURE 4:**
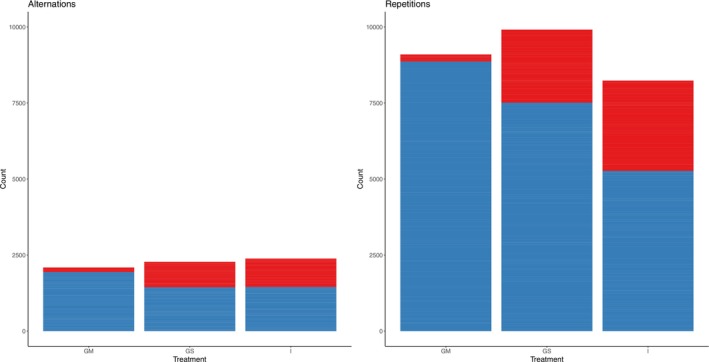
The number of alternations versus repetitions within treatments and sex (red = female, blue = male). GM = group mixed‐sex, GS = group single sex, I = isolated individuals. Samples sizes were *n* = 61 for GM females, *n* = 137 for GM males, *n* = 118 for GS females, *n* = 146 for GS males, *n* = 101 for I females, and *n* = 131 for I males. See Table S1 for partial trigrams.

Lastly, we found that individuals showed a significantly greater tendency to walk in repetitive trigrams (‘repetitions’) (i.e., LLL, RRR) compared to alternating trigrams (i.e., LRL, RLR) (*Z* = 103.09, *p* ≤ 0.001) (Figure 3). When analysing repetitive trigrams and alternating trigrams separately for ease of interpretation (note that we also detected a three‐way interaction between trigram type, sex, and treatment; see Table S1), we show that there is a significant sex × treatment interaction for both repetitive and alternating trigrams (Figure 4; Table 1; see Table S2 for an analysis of partial trigrams). Females performed the least alternation or repetition trigrams (i.e., moved the least), and females from GM treatment performed the least trigrams (both repetitive and alternating) compared to the other treatments (Figure 4; Table 1). When focussing on repetitive trigrams, males from the group mixed sex (GM) treatment performed the most repetitive trigrams, and males from the isolated (I) treatment performed the least repetitive trigrams (Figure 4). The opposite pattern occurred in females; females from the group mixed sex (GM) performed the least repetitive trigrams, and females from the isolated (I) treatment performed the most repetitive trigrams (Figure 4). Differences between individuals explained 43.71% of the total variation in alternation trigrams and differences between batches explained 1.32% of the total variation in alternation trigrams. Differences between individuals explained 21.02% of the total variation in repetitive trigrams, and differences between batches explained 3.01% of the total variation in repetitive trigrams.

### 3.2 Effects of Social Environment and Sex on Trait Correlations

**FIGURE 5:**
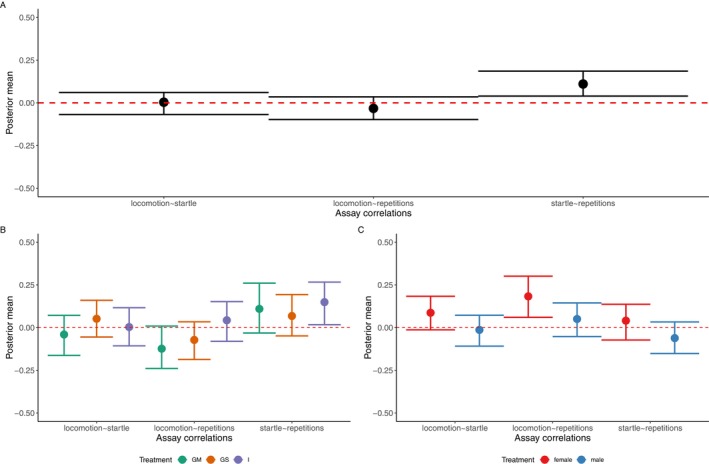
Posterior means and 95% Credible Intervals (CI) of the correlations between the average distance in the locomotion assay, startle response (binary), and number of repetitions in the y maze. (A) the overall correlations across treatment and sex, as well as grouped by (B) treatment and (C) sex. GM = group mixed sex; GS = group single sex; I = isolated individuals; loco = locomotion; reps = repetitions; startle = startle response.

We found that the startle response (i.e., if the flies showed a startle to the light‐off startle) and the number of repetitions were significantly positively correlated (Figure 5A; Table S3). However, the startle response and the number of repetitions did not correlate with locomotion (Figure 5A; Table S3).

When examining if correlations were only present under certain social contexts or within a particular sex, we found that only isolated individuals showed a significantly positive correlation between the startle response and the number of repetitions (Figure 5B; Table S3). We also found that females tended to show stronger positive trait correlations compared to males, where females, but not males, showed a significant positive correlation between startle response and repetitions and a positive, yet non‐significant, correlation between locomotion and startle response (Figure 5C; Table S3).

## 4 Discussion

Our study investigated the effects of social environment and sex on behaviours relating to exploration, habituation to stress, and memory in captive *Drosophila melanogaster* . We observed sex‐dependent effects of social stimulation on behaviour. Notably, we found that males tended to show greater exploration in both the locomotion assay and the y‐maze assay, but also that the amount of movement within the y‐maze (i.e., number of repetition and alternation trigrams) was dependent on the interaction between social environment and sex. We also found that females were more likely to show a habituation response and that the strength of this response was dependent on social environment. Lastly, in terms of cross‐trait correlations, females tended to have stronger trait associations than males, although we acknowledge that these results may need a larger sample size to achieve satisfactory power. Overall, our findings indicate that the social environment can affect behaviour in *Drosophila melanogaster* and that these behavioural responses can be sex‐dependent. This has implications for understanding the role of the social environment in shaping basic behaviours, particularly those that are likely to underlie more complex behaviours such as mate searching or foraging.

Behavioural plasticity in response to a range of external stimuli and across several traits has been reported in many species, including *Drosophila melanogaster* . Social cues are among some of the external factors that induce behavioural plasticity. Mated females can, for instance, alter their choosiness towards males via plastic changes in olfactory sensitivity to male pheromones (Kohlmeier et al. 2021). Similar responses were identified in males where perception of male–male competition plastically altered individual aggression levels (Nandy et al. 2016). Social proximity to competitors was also shown to modify male copulatory behaviour, even in species where remating is rare (i.e., where the risk of losing paternity to competitors should be absent; Lizé et al. 2011).

Our findings are also in line with previous research showing that males and females can respond differently to sensory and environmental stimulation (Fowler et al. 2019; Han et al. 2015; Videlier et al. 2019). Interestingly, we observed that males and females from the group mixed sex and isolated treatments showed opposite exploratory behaviour in the y‐maze. Males from the group mixed sex (GM) treatment showed the most exploration (both alternations and repetitions, although repetitions were considerably higher), while isolated males showed the least. In contrast, females from the GM treatment showed the least exploration, and isolated females showed the most (again, consistent between alternations and repetitions). The observed direction of differences aligns with published evidence on *D. melanogaster* y‐maze behaviour. For example, flies housed in intensely enriched environments (habitat enrichment with plants, artificial barriers and obstacles, large open space for exploration) tend to increase their exploratory behaviour in y‐mazes (Akhund‐Zade et al. 2019), both in terms of the number of turns and the variation in turning pattern. However, this study did not account for the potential sex differences that we have demonstrated. These differences may be related to sex‐specific effects of sensory stimulation incurred by social environment and/or differences in mating behaviour between the sexes. For example, social isolation can cause increased anxiety and reduced exploratory behaviour in other species (Mumtaz et al. 2018; Weiss et al. 2004). Nevertheless, the lack of social stimulation in the isolated treatment seemed to enhance exploration in females compared to those in the GM treatment. This sex difference in exploration in the GM treatment may be related to the refractory period experienced by previously mated females, where mated females will actively avoid additional matings, which may also correspond to reduced movement/exploration (Tompkins et al. 1982; Wolfner 1997). A reduction in exploratory behaviour in the isolated males was less pronounced, but movement within the Y‐maze was still substantially reduced compared to the GM (and, to a lesser extent, GS) treatments. In *D. melanogaster*, it is the male that exhibits active mating behaviour, where a lower exploratory tendency could reflect reduced social stimulation in the isolated group. Observed sex‐specificities may also partly reflect a weak inter‐sexual genetic correlation in sociality‐related behaviours (Scott et al. 2018), which would predispose such traits to independent evolution in opposite sexes.

Isolated individuals were more likely to show a startle response, and social treatment interacted with sex in how it affected the rate of habituation to the three consecutive light‐off startles. Part of this interaction resulted from males showing little to no such response, and females tending to show much stronger habituation in all social treatments (the slope of habituation response was particularly pronounced in the GM treatment). Such results are consistent with the rodent literature, where individuals with greater sensory stimulation through environmental enrichment show stronger habituation responses (Hughes and Collins 2010). Habituation also seems to depend on the stressfulness of the environment, decreasing in stressful conditions (Chouinard‐Thuly 2018). *D. melanogaster* seem to habituate relatively easily to stressful stimuli (e.g., chemical, mechanical or electric; see (Cho et al. 2004), and also (Engel and Wu 2009) for a brief review), but little is known about the sex‐specificity or plasticity of such responses. In particular, no studies exist in the context of social enrichment or deprivation.

Interestingly, we did not detect any effects of social environment or a sex treatment interaction on locomotory activity measured in the open arena setup (the locomotion assay). Both the y‐maze and movement within the well‐plates can be used to assess general exploratory behaviour (Simonnet et al. 2014; Cleal et al. 2021). Still, the y‐maze may provide a more realistic test of exploratory behaviour by allowing a larger suite of natural behaviours, such as turning and the use of short‐term working memory (Lewis et al. 2017). Alternatively, y‐maze assays may merge the effects of explorative behaviours with the general locomotory activity of flies (Buchanan et al. 2015). Even though we found that flies completed more repetition trigrams over alternation trigrams (a pattern confirmed also in (Cleal et al. 2021)), suggesting that they do not use strong working memories in this context, exploration within the y‐maze appears to allow for greater detection of social and sex‐specific effects compared to exploration within the simple well‐plate arena.

We also did not detect a correlation between the locomotory activity and the number of repetition trigrams within the y‐maze, apart from a weak negative correlation in the GM treatment. This suggests that these forms of exploration are not related and, in fact, may trade‐off with each other in previously mated individuals. Alternatively, locomotion within an open arena may not be representative of any natural conditions and should be revised as a behavioural assay. This result aligns with existing evidence showing little to no correlations between activity metrics and y‐maze behaviour in fruit flies at the between‐individual level (Werkhoven et al. 2021). We also did not detect a correlation between locomotion and the startle response even though these were conducted in the same arena. Werkhoven et al. 2021 also found no evidence for strong correlations between activity measures and phototaxis/optomotor handedness, which could be seen as distant analogues of our light‐off assay. However, we did detect a significant positive correlation between the startle response and the number of repetitions in the y‐maze, which was driven by females and isolated individuals. While it is unclear why these two responses are related under some contexts, these results show that both sex and social environment can affect some behavioural correlations. Notably, the observed sex effect (tendency for females to have stronger cross‐trait correlations) aligns with the published evidence. In *Drosophila serrata*, females tend to have stronger correlations between olfactory traits associated with mate choice (Gosden et al. 2012). Such patterns may reflect weaker direct and multivariate selection acting on females. It may also represent X‐linked genetic effects (*Drosophila* females are sexually diploid). However, a more detailed exploration of these correlations would require a richer quantitative genetic design and estimation of the between‐sexes cross‐trait covariances, the **B** matrix (Gosden et al. 2012). Further exploration of these patterns may be interesting in relation to potential behavioural syndromes (consistent within‐individual covariances of behavioural traits). Our estimates may be regarded as proxies of such syndromes but are almost certainly inflated estimates of them; studies with replicated assays performed on the same individuals (or multiple genotypes) are needed to decompose sources of variation into within‐ and between‐individual components (Dingemanse and Dochtermann 2013).

One important issue that applies to our study is the adequacy and biological interpretability of the assays we used. We decided to perform the specific assays for three main reasons. First, they represented the best trade‐off between the richness of the resulting data and the time constraint of each assay, allowing us to maximise the number of assayed flies. Second, the assays allowed for relatively simple transfer of assayed flies between different tests, enabling estimation of between‐individual correlations. Third, they were simple enough to facilitate their automation and higher throughput. The behavioural proxies of locomotion, exploratory behaviour, working memory, and stress habituation were also used previously in *Drosophila melanogaster* , yielding results comparable to published studies in terms of variability and magnitude of observed measurements (see, e.g., Fenckova et al. (2019); Cleal et al. (2021); Werkhoven et al. (2021)). Some may argue that the movement of animals in an open, circular arena or a narrow y‐maze has little biological relevance to natural locomotion and exploration patterns. However, it is generally assumed that standardised tests can measure consistent, repeatable components of more complex behaviours (Berman et al. 2014; Seidenbecher et al. 2020). Moreover, there is evidence that behaviour evolves modularly (Vinicius 2010; Eberhard 2018), that is, by selection acting on smaller units of more complex behaviours (Berman et al. 2014). Simplified assays can be seen as probing specific aspects of a behaviour (e.g., overall mobility, tendency to explore branching corridors) without getting drowned in random variation arising from other components responding to less controlled circumstances. Therefore, our assays still provide valuable information about dimensions of behavioural phenotypic space upon which ecological or evolutionary processes could act (Werkhoven et al. 2021).

Methodologically, alternatives exist that could be used in place of our phenotyping equipment (see, e.g., Werkhoven et al. (2019)). Still, we have no reason to suspect our approach would lead to any systematic biases in the measured parameters. One methodological difference that should be noted is that we avoided discarding flies that expressed activity below a certain threshold, as in some earlier studies (Buchanan et al. 2015; Werkhoven et al. 2021). The goals of our study are strongly focused on the evolutionary and ecological processes our study is assumed to represent. Thus, we are interested in the overall variation, which arguably should include low‐activity individuals. Both before and after each assay, we confirmed all flies were alive and active (clearly inactive individuals were identified and removed from analyses). Thus, the variation represented in this study is not biased by including defective/injured flies.

Understanding how the environment and sex shape basic behaviours is important as they can underlie more complex behaviours related to fitness and survival. For example, exploration and short‐term working memory are highly important for foraging and mate searching (Arenas et al. 2007; Lihoreau et al. 2009; Wilson et al. 2010). Additionally, startle responses are a good indicator of how an individual processes and responds to stimuli in their environments (Götz and Janik 2011; Hale et al. 2016; Sun et al. 2018). Overall, we show that the underlying behaviours related to these more complex behaviours can be sex‐dependent and shaped by the social environment. Further research investigating the genetic and plastic mechanisms underlying these responses will further enhance our understanding of the complex interplay between social environment, sex, and behaviour.

## Author Contributions

**Erin L. Macartney:** conceptualization (lead), data curation (equal), formal analysis (lead), investigation (lead), methodology (equal), resources (equal), validation (equal), visualization (equal), writing – original draft (lead), writing – review and editing (lead). **Samantha Burke:** investigation (supporting), validation (supporting), writing – review and editing (supporting). **Patrice Pottier:** data curation (supporting), investigation (supporting), validation (supporting), writing – review and editing (supporting). **Zina Hamoudi:** conceptualization (supporting), investigation (supporting), methodology (supporting), resources (supporting), writing – review and editing (supporting). **Chloe Hart:** investigation (supporting), resources (supporting), writing – review and editing (supporting). **Radiah Ahmed:** investigation (supporting), resources (supporting), writing – review and editing (supporting). **Yong Qi Lin:** investigation (supporting), resources (supporting), writing – review and editing (supporting). **G. Gregory Neely:** funding acquisition (supporting), methodology (supporting), resources (equal), supervision (supporting), writing – review and editing (supporting). **Szymon M. Drobniak:** conceptualization (equal), formal analysis (equal), funding acquisition (equal), investigation (equal), methodology (equal), supervision (equal), validation (equal), writing – review and editing (equal). **Shinichi Nakagawa:** conceptualization (equal), formal analysis (equal), funding acquisition (equal), investigation (equal), methodology (equal), project administration (lead), resources (lead), supervision (lead), validation (equal), writing – review and editing (equal).

## Conflicts of Interest

The authors declare no conflicts of interest.

## Supporting information


Appendix S1.

## Data Availability

Data and code is available through a Zenodo repository: https://zenodo.org/doi/10.5281/zenodo.13347772.
