# Supplementary material for: Sex‐Specific Effects of Social Environment on Behaviour and Their Correlations in Drosophila melanogaster
Source: Ecol Evol. 2025 Apr 25;15(4):e71261. doi: 10.1002/ece3.71261 (PMC12032194; doi:10.1002/ece3.71261)
Supplement: Supplementary file 1 — Appendix S1. [file ECE3-15-e71261-s001.docx]

**Supplementary material**

**Table S1** Analysis of the number of trigrams performed in the y-maze assay with a three-way interaction between trigram type, sex, and treatment included in the model.

|  | *χ^2^* | *p* |
| --- | --- | --- |
| Trigram type | **10283.78** | **<0.01** |
| Sex | **223.72** | **<0.01** |
| Treatment | 4.65 | 0.10 |
| Type x sex | **175.97** | **<0.01** |
| Type x treatment | **33.70** | **<0.01** |
| Sex x treatment | **159.75** | **<0.01** |
| Type x sex x treatment | **90.47** | **<0.01** |

**Table S2** Main effects and interactions of sex and treatment on the number of partial trigrams completed during the y-maze assay.

|  | *χ^2^* | *p* |
| --- | --- | --- |
| Sex | **186.56** | **<0.01** |
| Treatment | **8.53** | **0.01** |
| Sex x treatment | **131.49** | **<0.01** |

**Table S3** Posterior means and 95% Credible Intervals (CI) of the correlations between the average distance in the locomotion assay, startle response (binary), and number of repetitions in the y maze. Output shows the overall correlations across treatment and sex, as well as grouped by treatment and sex.

| Behaviour | Overall correlation | | By treatment | | | | | | By sex | | | |
| --- | --- | --- | --- | --- | --- | --- | --- | --- | --- | --- | --- | --- |
|  |  |  | GM | | GS | | I | | Female | | Male | |
|  | r | 95% CI | r | 95% CI | r | 95% CI | r | 95% CI | r | 95% CI | r | 95% CI |
| Locomotion~startle | <0.01 | -0.06, 0.07 | -0.04 | -0.16, 0.06 | 0.05 | -0.07, 0.14 | <0.01 | -0.11, 0.12 | 0.09 | -0.01, 0.17 | -0.01 | -0.11, 0.07 |
| Locomotion~repetitions | -0.03 | -0.10, 0.03 | **-0.12** | **-0.23,**  **-0.01** | -0.07 | -0.17, 0.05 | 0.04 | -0.05, 0.16 | 0.04 | -0.05, 0.15 | -0.06 | -0.16, 0.03 |
| Startle~repetitions | **0.11** | **0.04, 0.18** | 0.12 | -0.03, 0.25 | 0.07 | -0.05, 0.19 | **0.15** | **0.01, 0.27** | **0.18** | **0.07, 0.31** | 0.06 | -0.04, 0.16 |
